# Plant interactions, climate, and the reindeer (*Rangifer tarandus*) interdependently shape vegetation in northern Finland

**DOI:** 10.1002/eap.70200

**Published:** 2026-03-10

**Authors:** Sari Stark, Henri Wallén, Mika Kurkilahti, Antti‐Juhani Pekkarinen, Jouko Kumpula

**Affiliations:** ^1^ Arctic Centre, University of Lapland Rovaniemi Finland; ^2^ Natural Resource Institute Finland (Luke) Turku Finland; ^3^ Natural Resource Institute Finland (Luke) Helsinki Finland; ^4^ Natural Resource Institute Finland (Luke) Inari Finland

**Keywords:** boreal forests, *Cladonia* lichens, climate change, evergreen dwarf shrubs, range management, reindeer, subarctic ecosystems

## Abstract

There is limited understanding on how complex interdependencies among large herbivore grazing, competitive and facilitative interactions among plants, and the changes in temperatures and precipitation shape northern boreal and subarctic ecosystems. Here, we assessed changes in dwarf shrub and lichen cover and height using data from 617 field sites monitored a decade apart (2005–2008 and 2016–2018) in semi‐dry and dry habitats in northernmost Finland, where reindeer herding constitutes a traditional livelihood with reindeer moving freely across landscapes and grazing on seasonally available forage plants. We hypothesized that several direct and indirect factors related to climate and reindeer herding regimes drive changes in vegetation. We predicted that over the 10 years, shrub cover and height would increase and that lichen cover and height would decrease in response. We also expected that the decline in lichen cover and height would be more pronounced in summer‐grazed areas and in areas with higher reindeer densities. We observed that shrub cover and height had increased drastically, and the change in lichen cover was negatively correlated with change in shrub cover. However, the change in lichen height was positively correlated with change in shrub height, which reveals a dual relationship between dwarf shrub and lichen vegetation. The seasonal timing of reindeer grazing was also important: lichen cover decreased less in winter than summer and year‐round ranges. The direction of the change in lichen height was even opposite among seasonal ranges with decreased height in summer and year‐round ranges and increased height in winter ranges. Lichen cover and height responded negatively to higher reindeer densities in both summer and winter ranges. While shrub cover had increased in summer and year‐round ranges, shrub cover was unchanged in winter ranges, and shrub height increased less with increasing reindeer densities. These results indicate that reindeer grazing may partially counteract “shrubification” in areas that are grazed only during winter. Our results demonstrate how differing large herbivore grazing regimes, together with their complex interdependencies between climate warming and associated changes in plant–plant interactions, contribute to spatially variable vegetation trajectories. Due to the direct and the indirect mechanisms by which climate warming affects dwarf shrub and lichen vegetation, for maintaining good lichen grounds for reindeer herding, the benefit of seasonal range rotation will likely even increase in the future.

## INTRODUCTION

Climate change in the circumpolar Arctic is closely linked with increasing distribution and abundance of deciduous and evergreen shrubs (Bjorkman et al., 2020; Myers‐Smith et al., 2020), which has been mostly attributed to warmer growing season temperatures (Ackerman et al., 2018; Martin et al., 2017; Weijers et al., 2018). As an indirect consequence of intensified plant competition between vascular and non‐vascular plants, increase in shrubs parallels a decreased cover and biomass of ground lichens (Alatalo et al., 2017; Cornelissen et al., 2001; Fraser et al., 2014; Joly et al., 2009; Løkken et al., 2019), which typically constitutes a major component of the ground vegetation in semi‐dry and dry boreal and Arctic habitats. Evidence from the different parts of the Arctic shows that climate change shapes northern plant communities interactively with grazing by the reindeer (*Rangifer tarandus* L., caribou in northern America; Andruko et al., 2020; Odland et al., 2018; Skarin et al., 2020; Stark et al., 2021; Tuomi et al., 2024; Virtanen et al., 2010; Vowles et al., 2017; Zamin & Grogan, 2013), although also other herbivores exert important impacts (Barbero‐Palacios et al., 2024; Osterrieth & Bosker, 2024). It is currently viewed that grazing dampens the warming‐induced increase in deciduous tall and dwarf shrubs within a range of climatic conditions (Andruko et al., 2020; Bråthen, Ravolainen, et al., 2017; Olofsson et al., 2009; Post & Pedersen, 2008; Skarin et al., 2020; Spiegel et al., 2023; Vuorinen et al., 2022), but does not prevent the increase in evergreen shrub species like *Empetrum nigrum* L. (Bråthen et al., 2024; Bråthen, Gonzales, & Yoccoz, 2017; Maliniemi et al., 2018; Tuomi et al., 2024; Vowles et al., 2017; Vuorinen et al., 2017). It has also been suggested that decreased lichens through grazing may promote the establishment of trees and dwarf shrubs by removing the barriers for seedling germination (Sedia & Ehrenfeld, 2003; Tømmervik et al., 2004) and create a warmer soil microclimate due to the weaker insulation of the ground, which indirectly intensifies vegetation “shrubification” (Odland et al., 2018).

Observations on changes in vegetation from northernmost Fennoscandia largely confirm with these circumpolar trends (e.g., Bråthen et al., 2024; Maliniemi et al., 2018; Tuomi et al., 2024; Vuorinen et al., 2017). They heavily impact northern nature‐based livelihoods, such as reindeer (*R. tarandus* L.) herding, a traditional livelihood that relies on natural ranges with reindeer moving and grazing freely on various seasonally available foraging resources (Stark et al., 2023; Tuomi et al., 2024; Turunen et al., 2009). Reindeer utilize a diversity of vascular plant and lichen species as well as mushrooms in their diet (Bezard et al., 2015; Kojola et al., 1995; Storeheier et al., 2003), but the winter forage availability, particularly that of lichens, exerts a high importance for the survival and the body condition and weight of the reindeer (Kojola et al., 1995; Kumpula et al., 1998). Exclosure experiments conducted in dry, lichen‐rich boreal forests and subarctic tundra in northern Fennoscandia generally show a drastic increase in ground lichens in the absence of grazing (Akujärvi et al., 2014; den Herder et al., 2003; Köster et al., 2015; Olofsson et al., 2011; Stark et al., 2000; Tømmervik et al., 2012; Väre et al., 1995), thus demonstrating the role of reindeer grazing in shaping the lichen vegetation.

The simple assumption of the interactive role of warming and reindeer grazing in directing current northern vegetation trajectories leaves out several ecological factors known to affect shrub and lichen abundance. It is likely that the ongoing changes in climate intertwine with reindeer herding regimes in a complex fashion. Firstly, climate change does not solely result in increased temperatures, but also involves drastic changes in precipitation as well as the duration of the snow‐free period (Rasmus et al., 2020). While the length of the snow‐free period may benefit lichens that grow the most during autumn (Kytöviita & Crittenden, 2002), increasing dry periods during the growing season may reduce lichen growth, because lichens are physiologically active and photosynthesize only under conditions of sufficient moisture while staying dormant during drought (Jonsson Čabrajic et al., 2010, Jonsson Čabrajič et al., 2010; Lidén et al., 2010). Secondly, the effects of reindeer grazing on vegetation depend heavily on the seasonal timing of reindeer grazing (Kumpula et al., 2014; Stark et al., 2021; Sundqvist et al., 2019; Väisänen et al., 2014), which in turn are determined by the historical developments of each location (Helle & Jaakkola, 2008; Horstkotte et al., 2022; Stark et al., 2023). Grazing reduces lichens more strongly in summer‐grazed areas than in areas where reindeer can access only during winter, because the wintertime snow cover buffers the lichen cover against trampling (Kumpula et al., 2011, 2014; Stark et al., 2021). Increasing dry periods, in turn, substantially increase the sensitivity of lichens to trampling (Heggenes et al., 2017), which would then affect lichens in summer‐ but not in winter‐grazed areas. Further, despite the fact that it has long been considered that lichens are negatively impacted by increased dwarf shrubs (Alatalo et al., 2017; Cornelissen et al., 2001; Joly et al., 2009; Løkken et al., 2019), the role of changing plant–plant interactions under warming climate has not been previously incorporated into studies on the effects of reindeer herding on the current vegetation trends (e.g., Akujärvi et al., 2014; Kumpula et al., 2014; Miina et al., 2020).

To identify factors that underlie recent vegetation trends in northernmost Finland, we modeled changes in shrub and lichen cover and height in semi‐dry and dry habitats using data from 617 study sites from inventories conducted between the years 2005–2008 and 2016–2018 in northern boreal and subarctic ecosystems in Finland. The sites are rich in *Cladonia* L. lichens, expected to decrease in response to climate warming and grazing, and evergreen dwarf shrubs, such as *E. nigrum* and *Vaccinium vitis‐idaea* L., expected to increase in response to climate warming but respond weakly to grazing. We hypothesized that several direct and indirect factors related to climate and reindeer herding regimes, including plant competition, temperature, rainfall, reindeer densities, and the seasonal timing of grazing, interactively drive changes in shrub and lichen abundance. We predicted that (1) dwarf shrub cover and height have increased and lichen cover and height decreased during the study period and (2) the decrease in lichens is associated with the increase in shrubs. We further predicted that (3) the changes in shrubs are insensitive to grazing seasonality and reindeer densities, but lichens have decreased more in summer‐grazed areas and towards higher reindeer densities, and that (4) the changes in shrubs are positively influenced by higher growing season temperatures, whereas the changes in lichens are positively influenced by a higher number of rainy days during the growing season.

## METHODS

### Study area and vegetation analyses

The reindeer herding area extends to a land area of 122,936 km^2^, making over one‐third of the Finnish land area. Scots pine (*Pinus sylvestris* L.), Norway spruce (*Picea abies* (L.) H. Karst) and birch (*Betula* sp.) constitute the main tree species. Mountain birch (*Betula pubescens* ssp. *czerepanovii* (Orlova) Hämet‐Ahti) woodlands form the treeline ecotone with tundra heaths dominating at high altitudes and latitudes (Kuuluvainen et al., 2017; Virtanen et al., 2015). In semi‐dry and dry habitats located in mineral soils, dwarf shrubs such as crowberry (*E. nigrum*), bilberry (*Vaccinium myrtillus* L.), lingonberry (*V. vitis‐idaea*), and heather (*Calluna vulgaris* L.) dominate the field layer with *Cladonia* lichens and several moss species found in the ground layer.

Reindeer herding began in northern Finland during the 1600s. Reindeer herders generally protect lichen grounds from trampling through migrations between seasonal ranges (Horstkotte et al., 2022); however, in Finland, land borders prevent long seasonal migrations and herding has been organized within geographically defined herding districts since 1898, with fenced boundaries between the districts since about 1950s (Stark et al., 2023). Most herding districts practice year‐round grazing, where seasonal movements between landscapes, driven by forage availability and insect avoidance, occur within smaller distances scattered within the herding district (Kumpula et al., 2014). As a result, lichen abundances are lower than in other countries with similar or higher reindeer densities (Horstkotte et al., 2022). Many northernmost districts have established seasonal rotation during the late 1980s by bisecting districts into separate summer and winter ranges by fence (Kumpula et al., 2011; Stark et al., 2021). In the herding practices, calf slaughter, supplementary winter feeding and parasite treatment were adopted during late 1980s (Helle & Kojola, 1993; Kumpula et al., 2002). During the 1950s and 1960s, the total number of reindeer in Finland before annual slaughter was 120,000–160,000 reindeer, reaching a temporary historical maximum of over 250,000 reindeer during late 1980s and early 1990s, after which the winter herd stabilized to 180,000–200,000 reindeer for the last decades (Statistics of the Reindeer Herders' Association).

For monitoring changes in the lichen ranges, a site network for range inventories was established in dry and semi‐dry habitats in 1995 by the Finnish Game and Fisheries Research Institute, which later merged to form the Natural Resource Institute Finland (Luke). The site network includes 20 northernmost reindeer herding co‐operatives, 12 of which practices seasonal pasture rotation, that is, they have fenced areas for summer and winter grazing. On average, there are 31 monitoring sites per co‐operative. Here, we use vegetation data from two inventories conducted between 2005 and 2008, and between 2016 and 2018 (consequently referred to as the first and the second inventory). Each field site forms of a homogenous vegetation type along a straight measurement line (length 100 m) oriented either from south to north or from west to east. The start and the end points of the line are permanently marked in the field. Ten vegetation squares (size 0.5 × 0.5 m) located after every 10 m along the measurement line were analyzed by point intercept method using a square divided into 25 mesh points at which plants were observed (Moen et al., 2007). Despite measuring the distance between plots, their location might slightly deviate from each other between the inventories. Vascular plant, lichen and moss cover were calculated as the sum of intercepts observed in mesh points (one per species per mesh point). For reindeer lichens (*Cladonia stellaris*, *Cl. mitis*, *Cl. Rangiferina*, and *Cl. uncialis*) and dwarf shrubs, height also measured using a custom‐made scale pointed straight upwards at each grid point where they were observed. Of all the analyzed plant and lichen species, we used the following groups for data analyses: (1) dwarf shrubs (*V. myrtillus*, *V. uliginosum*, *V. vitis‐idaea*, *Calluna vulgaris*, and *E. nigrum*), (2) lichens (*Cl. stellaris*, *Cl. mitis*, *Cl. rangiferina* and *Cl. uncialis*), and (3) mosses.

### Statistical modeling

We employed Bayesian generalized hierarchical linear regression models to analyze changes in lichens and dwarf shrubs over time. We adopted a multivariate modeling approach (i.e., the model includes multiple response variables) that analyses four response variables simultaneously: lichen cover change, lichen height change, shrub cover change, and shrub height change, each with its own outcome distribution. These response variables were calculated at the plot level as differences between the first and the second inventories: *Y*_change_ = *Y*_*i*,second_ − *Y*_*i*,first_, which yielded 6170 observations. We applied a common set of explanatory variables across all response variables, which reflect three different types of ecological factors: (1) plant–plant relationships, (2) reindeer grazing intensity and seasonality, and (3) climatic conditions (explained in detail below). To account for the initial baseline, each response also included a unique baseline variable, which was the corresponding value from the first inventory (model structure summarized in Table 1).

**FIGURE 1:**
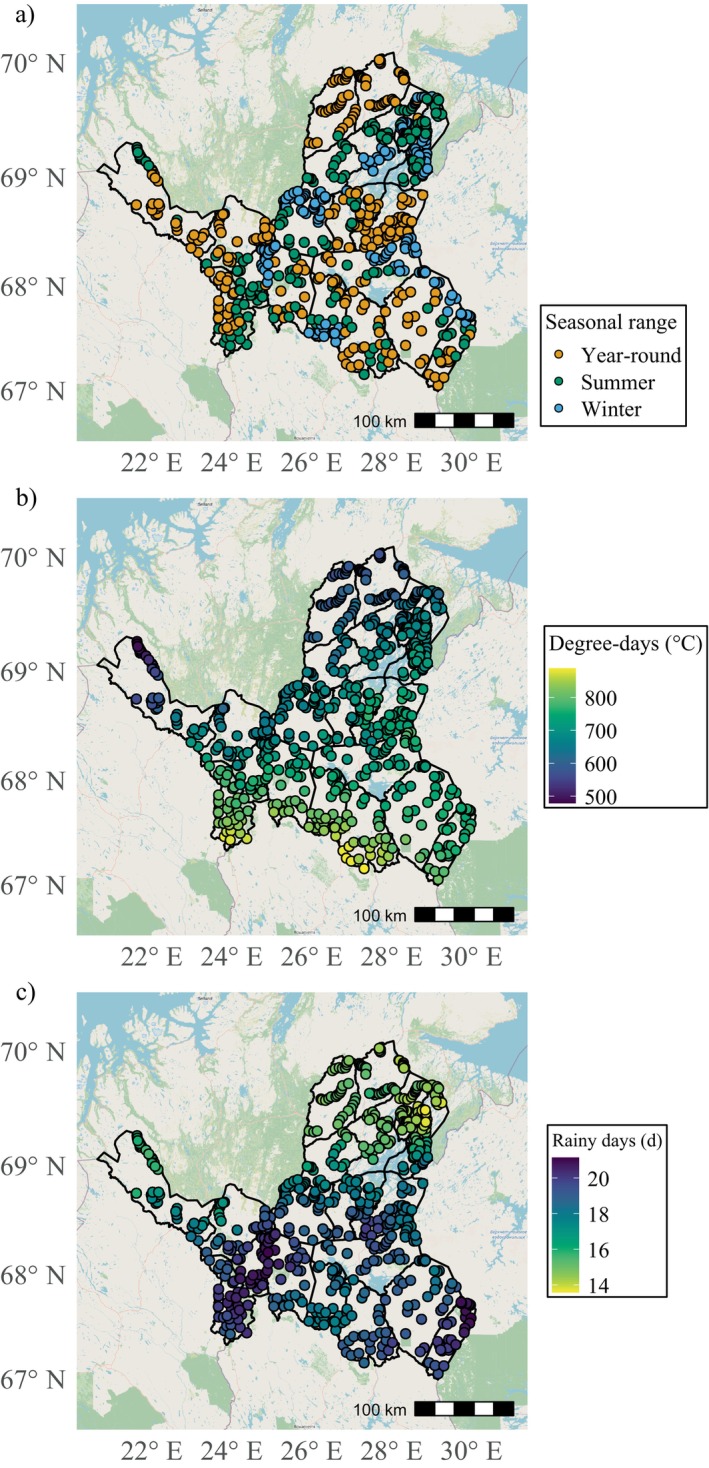
(a) Classification of grazing seasonality within each study site, (b) growing degree days (GDD5), and (c) the number of rainy days from the past 30 years.

1. Increased competition between dwarf shrubs and lichens is considered a major factor behind the circumpolar decrease in lichens (e.g., Alatalo et al., 2017; Cornelissen et al., 2001; Fraser et al., 2014). The multivariate framework allowed us to model potential plant–plant relationships by capturing residual correlations among the four response variables (lichen cover, lichen height, shrub cover, and shrub height) through a shared site‐level random effect. To account for competition between mosses and lichens (e.g., Tonteri et al., 2016; Väre et al., 1995), moss cover was included as an explanatory variable. We added the canopy cover% for each study site into the model to control the role of tree canopy, which regulates light availability for understory vegetation with important indirect effects on lichens (Jaakkola et al., 2013; Kumpula et al., 2014). We did not investigate the effects of forestry or other land uses as these will be studied separately in another context.
2. Each field site was assigned to one of the following classes depending on the seasonal timing of grazing: (1) winter range, (2) summer range, and (3) year‐round range (TOKAT Database, Finnish Reindeer Herders Association; Figure 1a). To describe reindeer grazing intensity, the average reindeer density in the overwintering herd from the past 10 years (Finnish Reindeer Herders Association Statistics) was included. Most herding districts include a high proportion of mesic habitats and peatlands used as summer ranges (Kumpula et al., 2019), especially in many geographically large districts. Calculating reindeer densities per total land area thus does not depict the grazing pressure on ground lichens in semi‐dry and dry habitats (Kumpula et al., 2014). We first calculated the area of lichen‐rich habitats per each reindeer co‐operative using satellite images (Appendix S1: Supplementary Methods), and then calculated reindeer densities per lichen range. As earlier studies have either found no relationship between reindeer densities and vegetation changes (Tuomi et al., 2024) or have found this relationship to be non‐linear (Sandström et al., 2016), reindeer densities were modeled as penalized thin plate splines (Pedersen et al., 2019; Wood, 2017) with coefficients that varied according to seasonal grazing classes. Splines do not assume any specific form for the relationship and are therefore flexible in modeling potentially complex, nonlinear patterns in the data, and therefore, this approach allows the data to determine the shape of the relationship without imposing strong assumptions.
3. To account for the climate changes, we used ClimGrid (The Finnish Meteorological Institute) that provides daily observations of climatic variables in a 10 × 10 km grid (Aalto et al., 2016). We calculated weather variables for each site as an average of 30 years prior the most recent inventory, using the exact year of monitoring (between 2016 and 2018) as the reference point. A long time period was selected as this would be less sensitive to the effect of exceptional years. As increased shrubs are attributed to warmer growing seasons (Ackerman et al., 2018; Martin et al., 2017; Weijers et al., 2018), we included growing degree days (GDD5) from the past 30 years as an explanatory variable (Figure 1b). We did not use the change in GDD5 during the study period, as climate has warmed so evenly across the study area (an increase of 50–60 annual degree days per decade; Rasmus et al., 2020). As the same air temperatures lead to higher soil temperatures in summer‐grazed areas (Odland et al., 2018; Stark et al., 2015), we included the interaction between GDD5 and grazing seasonality into the model. We also included the number of days during growing season when daily precipitation sum was over 5 mm as an explanatory variable (consequently referred to as rainy days; Figure 1c) for two reasons. Lichens are physiologically active only when moist (Jonsson Čabrajic et al., 2010, Jonsson Čabrajič et al., 2010; Lidén et al., 2010), and rainy days may thus positively influence the potential for lichen growth. Conversely, dry lichens are particularly susceptible for trampling (Heggenes et al., 2017), but as this would mostly affect lichens in summer and year‐round ranges, we included the interaction between rainy days and grazing seasonality into the model.

**TABLE 1:** The multivariate model structure for analyzing changes in lichen cover, lichen height, shrub cover, and shrub height between the years 2006–2008 (the first inventory) and the years 2016–2018 (the second inventory) in northernmost Finland.

Model element	Description	Factor
Response variables	Response variables (plot‐level differences between second and first inventories) are the dependent outcomes included in the multivariate model.	Lichen Cover; Lichen Height; Shrub Cover; Shrub Height
Fixed effects	Fixed effects refer to categorical explanatory variables	Seasonal range
Covariates	Covariates refer to continuous explanatory variables. Unique baseline variables are specific to each of the four response variables and are the corresponding value during the first inventory	Unique Baseline Variable; GDD5; Moss Cover Change; Rainy days; Reindeer density (spline); Tree canopy
Interaction terms	Interaction terms denote the modeled interactions between explanatory variables	Seasonal range × GDD5; Seasonal range × Rainy days; Seasonal range × Reindeer density
Random effect	Random effects describe hierarchical structure, with random intercepts calculated at the site level, nested within herding co‐operatives. Correlations among the response variables were estimated through a shared site‐level random effect.	(1 | district: site)

The models were fitted using the brms package (Bürkner, 2017) in the R programming environment (R Version 4.4.2) and implemented through Stan (Stan Development Team, 2023). Model assessment was conducted using the “leave‐one‐out” cross‐validation method (loo‐cv), utilizing the “loo” package (Vehtari et al., 2017) for the implementation. The computational analyses for the models were executed on the Puhti High‐Performance Computing (HPC) cluster, managed by CSC—the IT Center for Science. We utilized hierarchical linear models with a Student‐*t* distribution and an identity link function. We opted for the Student‐*t* distribution over the normal distribution due to its enhanced robustness in linear regression contexts. The Student‐*t* distribution is known for its ability to better accommodate outliers and data with thicker tails than the normal distribution (Gelman & Hill, 2006). The model architecture incorporated a nested random effect of study sites within a reindeer herding district (Table 1).

For the population‐level coefficients of each response variable, we employed normal priors with a mean (μ) of 0 and a SD (σ) of 0.25. The SDs of the group‐level random effects for each response variable were modeled using exponential priors with a rate (β) of 1. Additionally, to account for correlations between the group‐level effects across different response variables, we used a Cholesky LKJ correlation distribution with an eta value of 4. For an overview of the prior definitions, see Appendix S1: Table S1. The models were fitted using a series of 10 Markov Chains, each undergoing a total of 3750 iterations including 1250 for warm‐up. The R‐hat statistic consistently showed a mean of 1.00 across all parameters, indicating reliable model convergence. For effective sample sizes, Pareto diagnostics from loo‐cv and trace plots for chain convergence on key parameters, see Appendix S2: Tables S2–S5; Figure S1.

## RESULTS

### The changes in vegetation cover and height over the 10‐year study period

The absolute values for average shrub cover and height increased between the first and the second inventory (Table 2). The most common outcome of the change in shrub cover was an increase between 5% and 40%, whereas the changes in shrub height were highly variable across sites with a relatively even distribution of both increases and decreases across sites. *E. nigrum* constituted 38.6% and 43.2% *V. vitis‐idaea* 24.7% and 22.5%, *V. myrtillus* 18.2% and 15.4%, *C. vulgaris* 16.1% and 15.5%, and *V. uliginosum* 2.5% and 3.4% of the dwarf shrubs during the first and the second inventory, respectively. The average lichen cover had declined overall, and across the different sites, lichen cover had most commonly decreased by 5–40% (Table 2). *Cl. mitis* constituted 31.6% and 35.2%, *Cl. uncialis* 33.0% and 29.3%, *Cl. rangiferina* 16.9% and 24.5%, and *Cl. stellaris* 18.5% and 11.0% of the lichen cover during the first and the second inventory, respectively. On an average basis, the change in lichen height was close to zero; however, this mainly resulted from the fact that the different sites showed both increasing and decreasing trends.

**TABLE 2:** Lichen and dwarf shrub cover (hits in 0.5 m^2^ measurement plots per 100 mesh points) and height (in millimeters) in Finnish reindeer range inventory sites in 2008 and 2018.

	Year 2008	Year 2018	Changes among individual sites (percentage of sites)				
			Strong decrease	Decrease	Non‐changed	Increase	Strong increase
Lichen cover							
Year‐round range	24.3 (0.3)	17.9 (0.3)	1.1	46.4	41.4	11.1	*…*
Summer range	20.3 (0.4)	14.5 (0.3)	1.6	47.1	42.2	9.1	…
Winter range	29.9 (0.4)	23.5 (0.4)	*…*	53.3	34.9	11.8	*…*
Lichen height							
Year‐round range	14.0 (0.2)	13.7 (0.2)	8.8	39.5	12.3	23.0	16.5
Summer range	12.8 (0.2)	12.4 (0.3)	15.0	32.1	7.0	18.7	27.3
Winter range	18.6 (0.3)	18.7 (0.3)	5.3	39.6	7.7	21.9	25.4
Shrub cover							
Year‐round range	25.1 (0.4)	32.8 (0.4)	*…*	10.0	29.5	60.2	0.4
Summer range	28.8 (0.5)	35.6 (0.5)	*…*	21.9	25.7	51.3	1.1
Winter range	32.0 (0.5)	34.2 (0.5)	*…*	28.4	33.7	37.9	*…*
Shrub height							
Year‐round range	47.3 (0.6)	55.7 (0.6)	1.1	23.8	11.1	36.4	27.6
Summer range	58.5 (0.8)	65.4 (0.7)	2.7	34.2	14.4	25.1	23.5
Winter range	59.8 (0.7)	65.0 (0.7)	*…*	35.5	16.0	29.0	19.5

*Note*: Values are mean and SE in parentheses; *N* = 10 per site, 617 sites of which 261 are year‐round ranges, 187 are summer ranges, and 169 are winter ranges. The direction and the magnitude of changes per individual sites (non‐changed <5% change between inventories, decrease and increase between 5% and 40% change between inventories, strong decrease and strong increase >40% change between inventories).

### Bayesian modeling results

**FIGURE 2:**
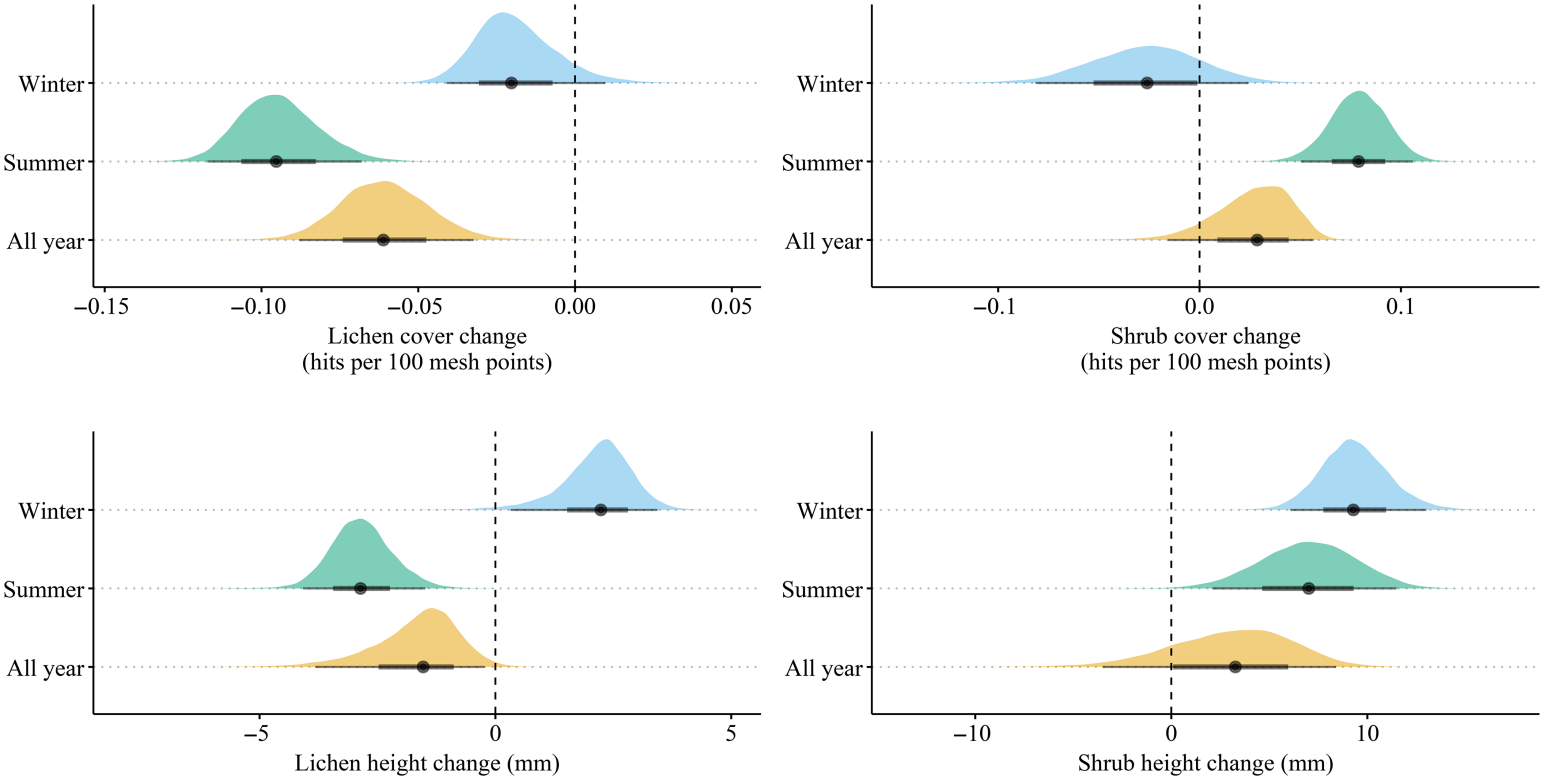
The posterior estimates for grazing seasonality on the expected change in lichen cover, shrub cover, lichen height, and shrub height.

The posterior estimate for the expected change in lichen cover was negative in all seasonal ranges, but the change was more negative in summer than winter ranges, with year‐round ranges between them (Figure 2, Appendix S3: Table S1). The direction of the change in lichen height varied depending on grazing seasonality: the posterior estimate for the expected change in lichen height was positive in winter ranges, but negative in summer and year‐round ranges. The credible interval for the change in shrub cover was large, but overall, posterior estimates for the expected change in shrub cover indicated a positive change in summer and year‐round ranges, but a slightly negative or a neutral change in winter ranges (Figure 2, Appendix S3: Table S1). The posterior estimates for the expected change in shrub height, in turn, indicated an increase in shrub height independent of grazing seasonality.

**FIGURE 3:**
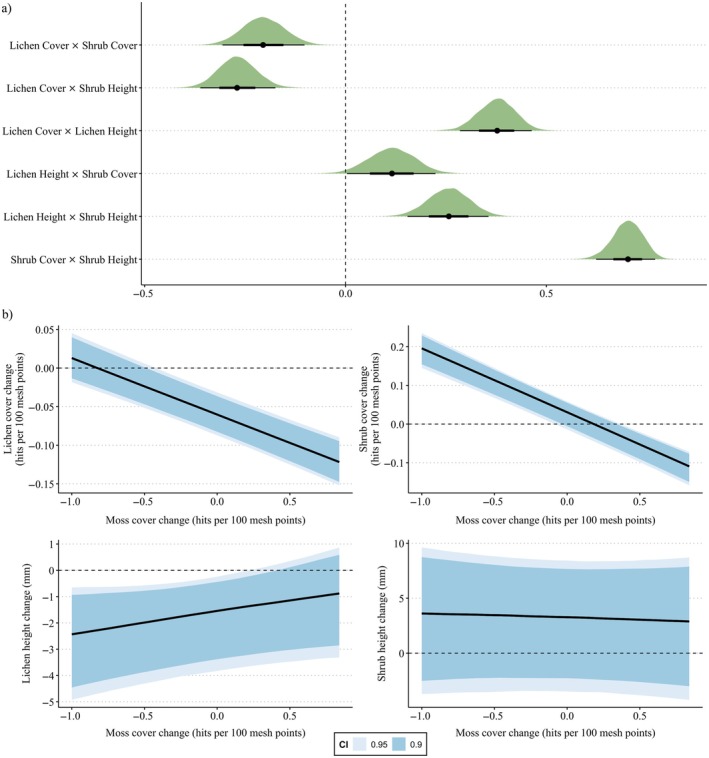
(a) Relationships among the changes in lichen cover, shrub cover, lichen height, and shrub height. (b) The relationship between the change in lichen cover and height with the change in moss cover.

The relationships between the changes in lichens and shrubs varied among response variables. There was a negative relationship between the changes in shrub cover and height with the change in lichen cover, that is, lichen cover had declined the most in sites where the shrub cover and height had increased the most (Figure 3a, Appendix S3: Tables S1–S4). In contrast, modeling indicated that the relationship between changes in lichen height and shrub cover and height was positive, that is, the change in lichen height was the most positive in sites with the greatest increase in shrub cover and height. The change in moss cover was negatively associated with the change in lichen and shrub cover, but there was no relationship with the changes in lichen and shrub height (Figure 3b, Appendix S3: Tables S1–S4). None of the relationships between shrubs, lichens and mosses varied among seasonal ranges (results separately per seasonal range not shown).

**FIGURE 4:**
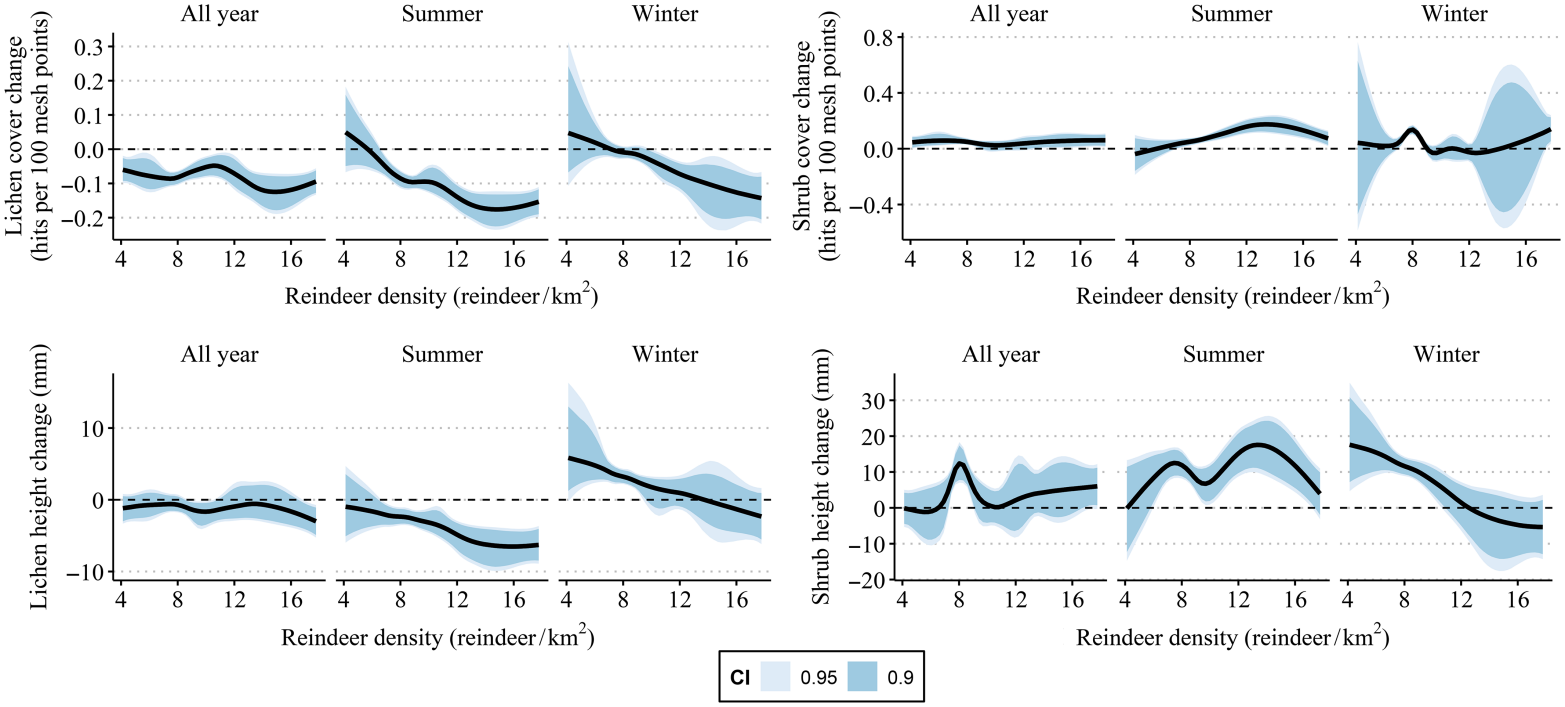
Relationships between reindeer densities (number of reindeer/lichen range area) with the change in lichen cover, the change in lichen height, the change in shrub cover, and the change in shrub height.

The credible intervals for the relationship between vegetation change and reindeer densities were large for many variables, especially in winter ranges. For the change in lichen cover and lichen height, modeling indicated a negative relationship with reindeer densities in both summer and winter ranges, but not year‐round ranges (Figure 4, Appendix S3: Tables S1–S4). There were no clear associations between reindeer densities and the changes in shrub cover and height, except for the change in shrub height that showed a negative relationship with reindeer densities in winter ranges.

**FIGURE 5:**
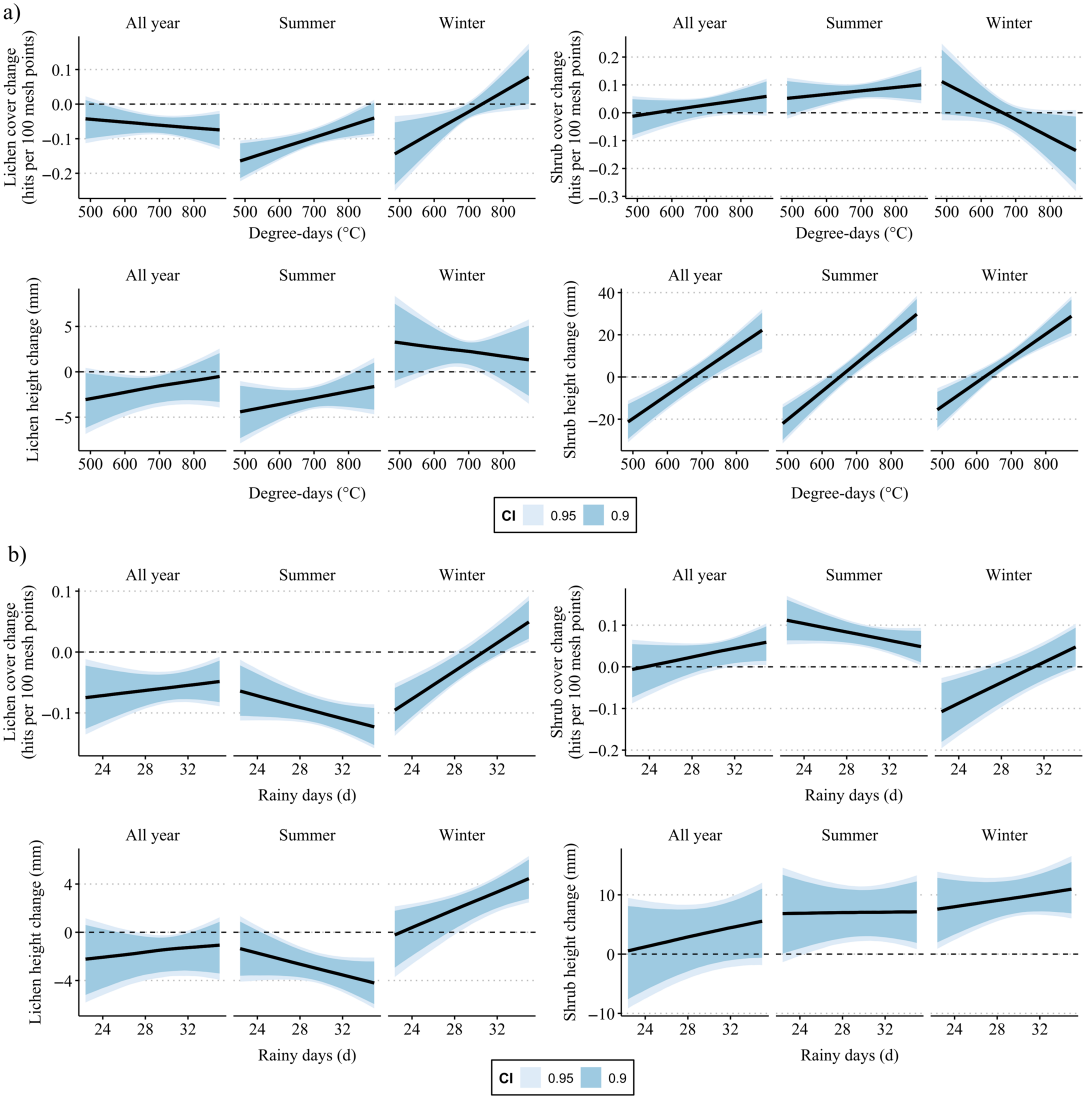
(a) Relationships between growing season temperature sum and (b) the number of rainy days during the growing season with the changes in lichen cover, lichen height, shrub cover, and shrub height.

Modeling indicated a positive association between GDD5 and changes in lichen cover in summer and winter ranges, but not in year‐round ranges. No associations were found between GDD5 and the changes in lichen height and shrub cover, but the change in shrub height was strongly and positively associated with GDD5 with the largest increase in height observed at the highest GDD5 values independent of grazing seasonality (Figure 5a, Appendix S3: Tables S1–S4). Modeling indicated no associations with rainy days, apart from the change in lichen cover, which showed a strong positive relationship in winter ranges but not in summer and year‐round changes (Figure 5b, Appendix S3: Tables S1–S4).

## DISCUSSION

### Do increasing evergreen dwarf shrubs compete with or facilitate the ground lichens?

Consistent with our first and fourth prediction, respectively, evergreen dwarf shrubs had increased during the 10‐year timeframe with the change in shrub height showing a strong linear positive relationship with GDD5. Shrub height had increased to a stronger extent than shrub cover, which agrees with findings by Tuomi et al. (2024) who found over a 20‐year timeframe in northernmost Norway a 14% increase in evergreen shrub cover but as high as 60% increase in biomass. In support for our second prediction that increased shrubs contribute to a circumpolar lichen decline (Alatalo et al., 2017; Cornelissen et al., 2001; Joly et al., 2009; Løkken et al., 2019), we found a negative relationship with changes in shrub and lichen covers. It is considered that shrubs gain a competitive advantage over lichens under a warmer climate and subsequently prevent lichen regeneration through increased shading (Fraser et al., 2014; Klein & Shulski, 2011). In line with earlier studies in Finland (Tonteri et al., 2016), we found a similar inverse relationship also between the changes in moss and lichen cover. Earlier studies have attributed the observed lichen decline to grazing intensity and seasonality added with the effects of forestry (Akujärvi et al., 2014; Jaakkola et al., 2013; Kumpula et al., 2014; Miina et al., 2020; Sandström et al., 2016) without considering ecological interactions among plants. As demonstrated here, assuming vegetation changes to be mediated only by external factors without considering drivers that operate within plant communities leaves an important gap in the identification of factors that underlie vegetation trends.

The second prediction assumed that the relationship between the changes in shrubs and lichen height would not differ from the relationship with the lichen cover. However, modeling indicated a synergistic relationship between lichen and shrub height, which contrasted this assumption. The non‐parallel patterns in lichen cover and height, firstly, reveal a dual relationship between the dwarf shrub and the ground lichen vegetation, in which shrubs may simultaneously both facilitate and compete with the lichens. Positive interactions among plants have a well‐demonstrated role in northern plant community dynamics, as a thick plant cover provides protection from temperature and moisture fluctuations and by this way, promotes the growth of subordinate species (e.g., Olofsson et al., 1999; Pellissier et al., 2010). We are, however, not aware of earlier studies that considered positive plant interactions in the context of ground lichens. In addition to a more favorable microclimate, lichens inside a thick and tall shrub cover may also be less likely to be consumed by the reindeer, as reindeer select for vegetation patches with the highest lichen coverage when foraging, especially from underneath the snow cover (Kumpula, 2001). Secondly, unsynchronized changes in lichen cover and height show that the characteristics of the ground lichen vegetation are currently undergoing a fundamental change, which should be integrated into the predictions for future vegetation trajectories as well as the management of the lichen grounds for reindeer herding.

### Complex interactions between reindeer grazing regimes with the direct and the indirect mechanisms by which climate change shapes vegetation

Consistent with our third prediction and the view that reindeer do not counteract vegetation “evergreening” (e.g., Bråthen et al., 2024; Bråthen, Gonzales, & Yoccoz, 2017; Bråthen, Ravolainen, et al., 2017; Tuomi et al., 2024; Vowles et al., 2017), overall, the changes in shrub cover and height did not depend on reindeer. However, in contrast to increased shrub cover in summer and year‐found ranges, in the winter ranges, the expected change in shrub cover was neutral or slightly negative. Further, in the winter ranges, the change in shrub height was negatively associated with reindeer densities. These results support findings that reindeer to some extent do limit vegetation “evergreening,” but that this phenomenon may be limited to important reindeer winter ranges (Finne et al., 2023; Stark et al., 2021), likely because evergreen dwarf shrubs constitute a considerable part of their winter diet (Heggberget et al., 2002; Kojola et al., 1995; Ophof et al., 2013). By contrast, in the summer ranges, increased soil temperatures (Odland et al., 2018) and a higher nitrogen availability (Stark et al., 2007) may promote *E. nigrum* growth (Aerts, 2010; Zamin et al., 2014). On the other hand, a total absence of grazing may benefit dwarf shrubs, as when undisturbed, lichens gradually build a thick layer of lichen necromass and soil organic matter, which increases soil moisture and water‐holding capacity, over time leading to a higher growth of vascular plants in dry habitats (Gaare, 1997).

In the associations between reindeer grazing and vegetation change, the patterns in the changes in lichen cover were a mirror image of the changes in shrub cover. Agreeing with the third prediction and similar to what has been earlier (Kumpula et al., 2014), the expected decline in the lichen cover was lower in winter ranges than in summer and year‐round ranges. However, contrasting with the overall decrease in lichen cover, the overall change in lichen height was relatively neutral across all study sites, and the expected change in lichen height was positive in winter ranges. Few studies have previously reported lichen height separately from cover or biomass, and in these studies, no such contrasting patterns between lichen cover and height were detected. Rather, these studies concluded that lichen height is a reliable yet simple and easily applicable measure for assessing reindeer grazing pressure on lichens (Olofsson, 2011; Olofsson et al., 2011). We find particularly noteworthy that, in winter ranges, the lichen layer had reduced in cover even when lichen height had simultaneously increased, that is, under a vegetation change that could be interpreted as lichen recovery from grazing. This calls for reassessing which indicator best reflects the grazing pressure in an environment currently undergoing multiple environmental changes and a transformation of composition, structure, and function (e.g., Antão et al., 2022).

The average reindeer density within the total area of the herding district does not catch the small‐scale spatial and temporal variation in grazing intensity across landscapes (e.g., Moen et al., 2009), and thus provides a crude estimation for grazing intensity for each site. Yet, consistent with our second prediction, the change in lichen cover was negatively associated with reindeer densities, although this relationship was not visible in year‐round ranges. Noteworthy, the change in lichen cover was negative across the entire range of reindeer densities in both summer and winter ranges. Only the change in lichen height in winter ranges showed an increase over time at the lowest reindeer densities. Interestingly, in the winter ranges, the change in lichens also showed a synergistic relationship with the number of rainy days, that is, conditions most favorable for new growth (Jonsson Čabrajic et al., 2010, Jonsson Čabrajič et al., 2010; Lidén et al., 2010), which supported the fourth prediction while also demonstrating that the role of favorable growing conditions for the lichens can be witnessed only in areas where the physical consumptive mechanisms, such as trampling, have a weaker role. Put together, these findings highlight how challenging it would be, under current environmental conditions, to increase lichen availability by manipulating reindeer densities alone (Pekkarinen et al., 2015).

### Implications for sustainable reindeer herding and forage availability under changing climate

**FIGURE 6:**
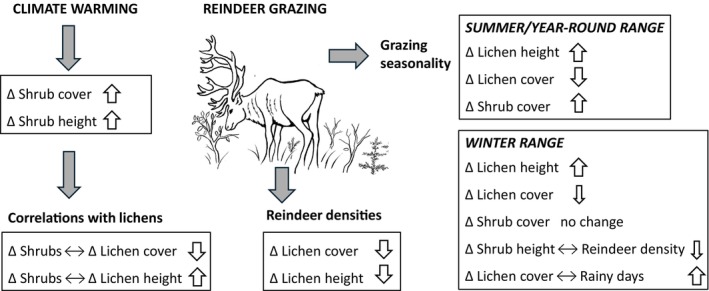
Graphical summary on how climate warming, altered interactions between dwarf shrubs and lichens, and the reindeer (*Rangifer tarandus* L.) drive changes (∆) in dwarf shrub and lichen cover and height. The symbol ↔ denotes a relationship between two factors. The thin arrows indicate weaker change over time than the thick arrows. Illustration credit: Jan Lipponen.

Vegetation changes induced by climate change have substantial ecological and socio‐economic consequences for reindeer herding in northern Fennoscandia (Nhat et al., 2024; Pekkarinen et al., 2015), which add to other current pressures in boreo‐arctic reindeer ecosystems, such as harsh winters and increasing infrastructures (Bjerke et al., 2024; Kumpula et al., 2024; Rasmus et al., 2020; Stoessel et al., 2022). Also forestry reduces lichen abundances both directly and in interaction with reindeer grazing particularly in Finland and Sweden (Jaakkola et al., 2013; Kumpula et al., 2014; Sandström et al., 2016; Tonteri et al., 2016). Earlier assessments using lichen biomass (as in Kumpula et al., 2014, 2019) may have even underestimated the lichen decline, because increased lichen height in some areas has compensated for the decreased lichen cover. Owing to these factors, the density of reindeer in which the quantity of lichen biomass would remain stable gets lower or may not even exist. According to simulations by Pekkarinen et al. (2020), the grazing capacity of ranges will likely further reduce in the future, but adapting to this change by reducing reindeer numbers alone (i.e., without considering seasonal range rotation or other land uses) may lead to an outcome where reindeer herding is no longer economically feasible. As reindeer herding represents a traditional livelihood in the Arctic area, and is an intrinsic part of the indigenous Sámi culture, this would be most alarming. Due to the interdependencies among climate, plant competition and the reindeer (Figure 6), under the warmer climate, seasonal range rotation is likely even more beneficial practice in reindeer herding than before. However, this is often difficult to implement in practice due to fragmented locations of different seasonal ranges, busy road networks, and infrastructures (Kumpula et al., 2019), and therefore, creating ways to overcome these obstacles would be a recommended course in range management. However, as our models showed decreased lichen cover even in the winter ranges and at low reindeer densities, our study also demonstrates a difficult challenge in maintaining good lichen grounds under the warming climate.

The same interdependencies among large grazers and climate‐induced vegetation trends detected here drive vegetation trends at the circumpolar level. Evidence exists from throughout the Arctic that the two major vegetation changes, that is, increasing shrubs and decreasing lichens, occur in an association with each other (e.g., Alatalo et al., 2017; Fraser et al., 2014). Studies from both Eurasian and American continents also indicate that once lost, lichens have great difficulties in re‐establishing (Gao et al., 2023; Tømmervik et al., 2004). Wild caribou populations in northern American continent utilize a wide range of different types of forage, but also there, lichens constitute the most important winter nutrition for *R. tarandus* (Webber et al., 2022). Although both negative and positive effects of the vegetation changes on the forage quantity and quality for caribou have been found (Richert et al., 2021; Zamin et al., 2017), some evidence suggests that changes in forage availability may contribute to the recent decline in caribou populations (Denryter et al., 2021). Due to the fundamental changes taking place in lichen‐rich vegetation types across the Arctic, there is a high need for protecting the remaining lichen grounds throughout the circumpolar north.

## AUTHOR CONTRIBUTIONS

Jouko Kumpula led field measurements and initial data processing. Jouko Kumpula, Sari Stark, and Mika Kurkilahti planned the study. Henri Wallén processed data and prepared all figures. Henri Wallén and Mika Kurkilahti conducted data modeling to which Jouko Kumpula, Sari Stark, and Antti‐Juhani Pekkarinen contributed with variable selection. Sari Stark led the writing of the paper to which all co‐authors contributed with discussion and text.

## CONFLICT OF INTEREST STATEMENT

The authors declare no conflicts of interest.

## Supporting information


Appendix S1.

Appendix S2.

Appendix S3.

## Data Availability

Data and code (Wallén et al., 2025) are available in Zenodo at https://doi.org/10.5281/zenodo.14643132. Study site locations are not disclosed to respect the privacy of the landowners and to negate any risk of outside interference with sites. Full data are available to qualified researchers upon reasonable request to Antti‐Juhani Pekkarinen (antti-juhani.pekkarinen@luke.fi).
