# Supplementary material for: Plant interactions, climate, and the reindeer (Rangifer tarandus) interdependently shape vegetation in northern Finland
Source: Ecol Appl. 2026 Mar 10;36(2):e70200. doi: 10.1002/eap.70200 (PMC12974556; doi:10.1002/eap.70200)
Supplement: Supplementary file 1 — Appendix S1. [file EAP-36-e70200-s001.pdf]

Plant interactions, climate, and the reindeer (*Rangifer tarandus*) interdependently shape vegetation in northern Finland

Authors: Sari Stark, Henri Wallén, Mika Kurkilahti, Antti-Juhani Pekkarinen and Jouko Kumpula

Ecological Applications

Appendix S1: Supplementary methods.

Different types of landscapes within the herding co-operatives were mapped using the semi-supervised interpretation method of satellite images, in which image and classification data were processed and corrected both before and following the image classification (Tanskanen, 2007). Similar methods have been used for pasture mapping in Norway (Johansen and Karlsen 2002; 2005; Johansen 2004). Additional details for the method can be found in Tanskanen (2007) and Kumpula et al. (2008).

Satellite images were first corrected to a unified coordinate system using ArcMap software. Non-relevant land features such as water bodies, peatlands, urban areas, and built environments were removed using digital topographic datasets to focus the analysis on relevant land cover types in uplands and mineral soils. The classification was performed using Erdas software, where pixels from the mapped area were first grouped into 100–130 classes based on their spectral properties. These spectral classes were then aggregated and merged into 30–50 intermediate land cover categories by cluster analysis. The final 19 vegetation classes were formed by further combining the similar type categories, using supporting datasets such as aerial photographs, topographic maps, the Lucas database (https://ckan.ymparisto.fi/dataset/lucas2018), field survey plots, and forest and vegetation type data. Peatlands were divided into open and forested categories using terrain classification

datasets from the National Land Survey of Finland. Drained peatlands were separated from other peatlands using Modelmaker, a tool of Erdas software.

Once all individual image classifications were completed, they were merged into a unified vegetation classification covering the entire reindeer herding area. Corrections were made after testing a classification by additional datasets, when it was evaluated necessary. The data set used for the classification the whole reindeer herding area of Finland consisted of 20 Landsat 5-TM images and 17 Landsat 8 images. The classified satellite images from 2002-2016, with the majority between 2006 and 2015. The 19 resulting vegetation classes were then grouped into nine main vegetation classes based on previous inventories (Kumpula et al., 1997, 2009). The classified data from different parts of the reindeer herding area were combined into a complete data set using the Erdas software. ArcMap software used this dataset to calculate the area of each vegetation class within a herding district, both for the 19-class categorization and the aggregated 9-class categorization.

In the final stage, reindeer co-operative boundaries were integrated into the analysis to enable area calculations for each class within individual reindeer co-operative. For obtaining a relevant measure of reindeer densities, the total number of reindeer in winter herd in each co-operative was then divided by the area of lichen-rich vegetation types.

References

Johansen, B. and S.R. Karlsen. 2002. "Finnmarksvidda – changes in lichen cover 1987–2000". *Rangifer Report* No. 6: 65–66.

Johansen, B. 2004. "Mountain vegetation mapping in Dovre area, Norway, using Landsat TM data and GIS." In: Ehlers, M., Kaufmann, H.J & Michel, U. (eds.) "Remote Sensing for

Environmental Monitoring, IS Applications, and Geology III”. *Proc. SPIE* Vol 5239: 333–344.

Johansen, B. and S. R. Karlsen. 2005. ”Rik lauvskog i Finnmark – undersøkelser av nye lokaliteter og oppdatering av tidligere vurderte lokaliteter. Fylkesmannen i Finnmark, miljøvernavdelingen”. Rapport nr. 1–2005. 58 sider.

Kumpula, J., A. Colpaert, and M. Anttonen. 2007. “Does forest harvesting and linear infrastructure change the usability value of pastureland for semi-domesticated reindeer (*Rangifer tarandus tarandus*)?” *Annales Zoologici Fennici* 44: 161–178.

Kumpula, J., A. Colpaert, and A. Tanskanen, A. 2008. “Porojen laidunten valinta muuttuneessa metsä- ja maisemarakenteessa Keski-Lapissa”. *Suomen Riista* 54: 69–82.

Kumpula, J., A. Tanskanen, A. Colpaert, M. Anttonen, H. Törmänen, J. Siitari, and S. Siitari, J. 2009: ”Poronhoitoalueen pohjoisosan talvilaitumet vuosina 2005–2008 – Laidunten tilan muutokset 1990-luvun puolivälin jälkeen.” *Riista- ja Kalatalous –Tutkimuksia* 3/2009: 1–48.

Kumpula, J., J. Siitari, S. Siitari, M. Kurkilahti, J. Heikkinen, and K. Oinonen, K. 2019. ”Poronhoitoalueen talvilaitumet vuosien 2016–2018 laiduninventoinnissa: Talvilaidunten tilan muutokset ja muutosten syyt”, 86 Pages. Luonnonvarakeskus (In Finnish).

Tanskanen, A. 2007: ”Satelliittikuvatulkintamenetelmät porolaidunkartoituksessa: puoliohjaamaton luokitus.” MSc Thesis, University of Joensuu, 63 pages and 14 appendices.
