# Supplementary material for: Plant interactions, climate, and the reindeer (Rangifer tarandus) interdependently shape vegetation in northern Finland
Source: Ecol Appl. 2026 Mar 10;36(2):e70200. doi: 10.1002/eap.70200 (PMC12974556; doi:10.1002/eap.70200)

Plant interactions, climate, and the reindeer (*Rangifer tarandus*) interdependently shape vegetation in northern Finland

Authors: Sari Stark, Henri Wallén, Mika Kurkilahti, Antti-Juhani Pekkarinen and Jouko Kumpula

Ecological Applications

Appendix S2: Priors and Model Diagnostics

Table S1. The summary of priors specified as a set of weakly informative priors for the parameters. For the population-level coefficients of each response variable, normal priors with a mean of 0 and a standard deviation of 1 were assigned. The standard deviations of the group-level random effects for each response variable were given exponential priors with a rate parameter of 1. Additionally, a Cholesky LKJ correlation distribution was used with an eta value of 2 to account for correlations between the group-level effects across different response variables.

Response	Effect	Class	Prior	Lower bound	Upper bound
Lichen Cover Change	Population	Beta	Normal(0, 1)		
Shrub Cover Change	Population	Beta	Normal(0, 1)		
Lichen Height Change	Population	Beta	Normal(0, 1)		
Shrub Height Change	Population	Beta	Normal(0, 1)		
Lichen Cover Change	Population	Intercept	Normal(0, 1)		
Shrub Cover Change	Population	Intercept	Normal(0, 1)		
Lichen Height Change	Population	Intercept	Normal(0, 2)		

Shrub Height Change	Population	Intercept	Normal(0, 2)		
Lichen Cover Change	Group	Standard deviation	Exponential(1)	0	
Shrub Cover Change	Group	Standard deviation	Exponential(1)	0	
Lichen Height Change	Group	Standard deviation	Exponential(1)	0	
Shrub Height Change	Group	Standard deviation	Exponential(1)	0	
All responses	Group	Correlation	LKJ(2)		
Lichen Cover Change	Distribution	Sigma	Exponential(1)	0	
Shrub Cover Change	Distribution	Sigma	Exponential(1)	0	
Lichen Height Change	Distribution	Sigma	Exponential(1)	0	
Shrub Height Change	Distribution	Sigma	Exponential(1)	0	

Model Diagnostics. The Markov Chain Monte Carlo (MCMC) and Leave-One-Out Cross-Validation (loo-CV) diagnostics of the Bayesian hierarchical linear regression model used for analysing the data set on the changes of lichen cover, lichen height, shrub cover, and shrub height between 2008-2018 in the northernmost Finland. The models were fitted using a series of 10 Markov Chains, each undergoing a total of 4000 iterations, of which 1000 for warmup. Effective sample sizes (ESS) for Population -level and group -level parameters are shown in Table S2, Standard Deviations and Correlations are shown in Table S3, $\hat{R}$ values are shown in Table S4, and smoothing Spline hyperparameters for modeling reindeer densities are shown in Table S5. These ESS values suggest efficient sampling and support robust statistical inferences. The $\hat{R}$ statistic consistently showed a mean of 1.00 across all parameters, indicating reliable model convergence. Model reliability was further verified through detailed visual inspections of trace plots from each chain, confirming appropriate mixing and stationarity for population and group -level parameters.

Table S2. Population-level effect parameters.

Response	Parameter	Level	$\hat{R}$	Bulk ESS	Tail ESS
Lichen Cover Change	Intercept		1.001	4443	8057
Lichen Cover Change	Seasonal Range	Summer	1.002	4246	7176
Lichen Cover Change	Seasonal Range	Winter	1.002	4334	7574
Lichen Cover Change	Lichen Cover 2008		1	17255	15780
Lichen Cover Change	Tree canopy pct 2018		1.001	4961	8562
Lichen Cover Change	Moss Cover Change		1.001	23071	16172

Lichen Cover Change	Rainy days		1.003	3939	7414
Lichen Cover Change	GDD5		1.002	3191	6682
Lichen Cover Change	Seasonal Range: Summer × Rainy days		1.003	3974	7074
Lichen Cover Change	Seasonal Range: Winter × Rainy days		1.002	4030	7909
Lichen Cover Change	Seasonal Range: Summer × GDD5		1.002	3772	6741
Lichen Cover Change	Seasonal Range: Winter × GDD5		1.002	4189	7131
Lichen Cover Change	Reindeer Density × Seasonal Range	All	1.002	5023	5412
Lichen Cover Change	Reindeer Density × Seasonal Range	Summer	1.001	7674	8965
Lichen Cover Change	Reindeer Density × Seasonal Range	Winter	1.001	6287	4874
Lichen Height Change	Intercept		1.001	6299	10797
Lichen Height Change	Seasonal Range	Summer	1.002	6598	10550
Lichen Height Change	Seasonal Range	Winter	1.001	9290	13082
Lichen Height Change	Lichen Height 2008		1	17117	16053
Lichen Height Change	Tree canopy pct 2018		1.002	5581	9295
Lichen Height Change	Moss Cover Change		1	22124	16480
Lichen Height Change	Rainy days		1.002	3831	7782
Lichen Height Change	GDD5		1.002	4242	8075
Lichen Height Change	Seasonal Range:		1.002	4207	7798

	Summer × Rainy days				
Lichen Height Change	Seasonal Range: Winter × Rainy days		1.002	3566	6983
Lichen Height Change	Seasonal Range: Summer × GDD5		1.003	4707	8255
Lichen Height Change	Seasonal Range: Winter × GDD5		1.001	5365	10389
Lichen Height Change	Reindeer Density × Seasonal Range	All	1.001	8383	8177
Lichen Height Change	Reindeer Density × Seasonal Range	Summer	1.001	7796	10255
Lichen Height Change	Reindeer Density × Seasonal Range	Winter	1	9861	8146
Shrub Cover Change	Intercept		1.002	6896	11273
Shrub Cover Change	Seasonal Range	Summer	1.001	6740	10976
Shrub Cover Change	Seasonal Range	Winter	1	7247	11595
Shrub Cover Change	Shrub Cover 2008		1	21637	15684
Shrub Cover Change	Tree canopy pct 2018		1.001	7414	12379
Shrub Cover Change	Moss Cover Change		1	23573	16263
Shrub Cover Change	Rainy days		1.002	4973	9474
Shrub Cover Change	GDD5		1.001	5425	9670
Shrub Cover Change	Seasonal Range: Summer × Rainy days		1.001	5027	8907
Shrub Cover Change	Seasonal Range: Winter × Rainy days		1.001	5470	9744

Shrub Cover Change	Seasonal Range: Summer × GDD5		1.002	5572	9558
Shrub Cover Change	Seasonal Range: Winter × GDD5		1.001	6318	10342
Shrub Cover Change	Reindeer Density × Seasonal Range	All	1.002	5813	5630
Shrub Cover Change	Reindeer Density × Seasonal Range	Summer	1	9641	11752
Shrub Cover Change	Reindeer Density × Seasonal Range	Winter	1.001	9994	12949
Shrub Height Change	Intercept		1	7306	11103
Shrub Height Change	Seasonal Range	Summer	1	15022	15860
Shrub Height Change	Seasonal Range	Winter	1.001	22289	15644
Shrub Height Change	Shrub height 2008		1	22056	16178
Shrub Height Change	Tree canopy pct 2018		1.002	7659	12345
Shrub Height Change	Moss Cover Change		1	23385	15774
Shrub Height Change	Mean Rainy days		1.001	4800	9041
Shrub Height Change	GDD5		1.001	3904	6198
Shrub Height Change	Seasonal Range: Summer × Rainy days		1.001	4724	8785
Shrub Height Change	Seasonal Range: Winter × Rainy days		1.001	5004	9544
Shrub Height Change	Seasonal Range: Summer × GDD5		1.001	4670	8101

Shrub Height Change	Seasonal Range: Winter × GDD5		1	5608	10406
Shrub Height Change	Reindeer Density × Seasonal Range	All	1	15726	14459
Shrub Height Change	Reindeer Density × Seasonal Range	Summer	1	18332	15580
Shrub Height Change	Reindeer Density × Seasonal Range	Winter	1.001	10898	12735

Table S3. Group -level standard deviation parameters (SD).

Response	Parameter	$\hat{R}$	Bulk ESS	Tail ESS
Lichen Cover Change	Intercept	1.001	5522	10572
Lichen Height Change	Intercept	1.001	5895	11313
Shrub Cover Change	Intercept	1.001	7832	13062
Shrub Height Change	Intercept	1.001	5416	11048

Table S4: Group -level correlation parameters (COR)

Response1	Response2	$\hat{R}$	Bulk ESS	Tail ESS
Lichen Cover	Lichen Height	1.002	4337	8289
Lichen Cover	Shrub Cover	1.001	5185	9522
Lichen Height	Shrub Cover	1.001	4951	9362
Lichen Cover	Shrub Height	1.001	4755	9523
Lichen Height	Shrub Height	1.001	5011	9202
Shrub Cover	Shrub Height	1.001	5542	10352

Table S5. Smoothing Spline Hyperparameters

Response	Parameter1	Parameter2	Level	$\hat{R}$	Bulk ESS	Tail ESS
Lichen Cover	Reindeer density	Seasonal Range	All-Year	1.002	2090	1969
Lichen Cover	Reindeer density	Seasonal Range	Summer	1.004	2197	3717
Lichen Cover	Reindeer density	Seasonal Range	Winter	1.003	2329	5246
Lichen Height	Reindeer density	Seasonal Range	All-Year	1.006	1787	5092
Lichen Height	Reindeer density	Seasonal Range	Summer	1.004	2201	3591
Lichen Height	Reindeer density	Seasonal Range	Winter	1.003	2464	2831
Shrub Cover	Reindeer density	Seasonal Range	All-Year	1.005	1957	5218
Shrub Cover	Reindeer density	Seasonal Range	Summer	1.001	5558	9118
Shrub Cover	Reindeer density	Seasonal Range	Winter	1.002	5352	7728
Shrub Height	Reindeer density	Seasonal Range	All-Year	1.002	2047	2119
Shrub Height	Reindeer density	Seasonal Range	Summer	1.002	3715	3335
Shrub Height	Reindeer density	Seasonal Range	Winter	1.002	4576	7321

Pareto statistic. The loo-package for computing Pareto k estimates and calculation of effective number of parameters (p_loo) was applied for each submodel. All Pareto k values were under 0.7, and p_loo values were lower than the total number of parameters, pointing to a well-functioning model.

Figure S1. PSIS diagnostic (LOO).

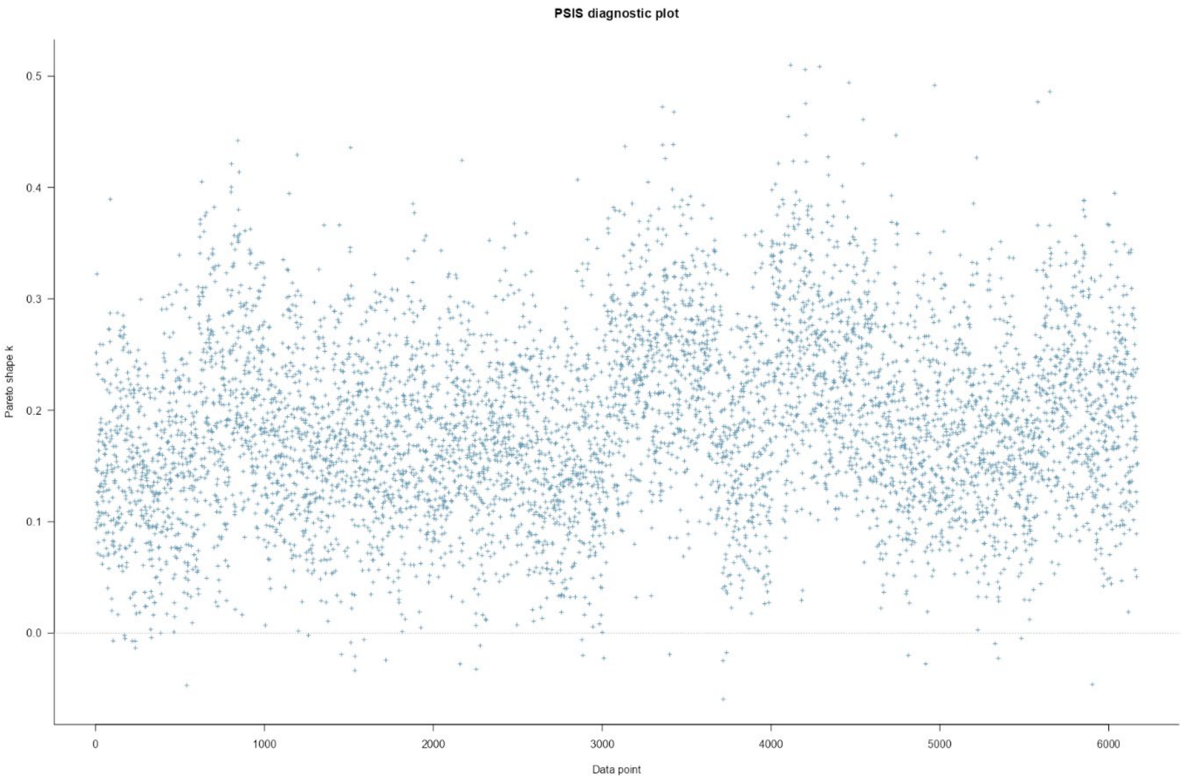

Supplement: Supplementary file 2 — Appendix S2. [file EAP-36-e70200-s002.pdf]
