# Supplementary material for: Plant interactions, climate, and the reindeer (Rangifer tarandus) interdependently shape vegetation in northern Finland
Source: Ecol Appl. 2026 Mar 10;36(2):e70200. doi: 10.1002/eap.70200 (PMC12974556; doi:10.1002/eap.70200)
Supplement: Supplementary file 3 — Appendix S3. [file EAP-36-e70200-s003.pdf]

Plant interactions, climate, and the reindeer (*Rangifer tarandus*) interdependently shape vegetation in northern Finland

Authors: Sari Stark, Henri Wallén, Mika Kurkilahti, Antti-Juhani Pekkarinen and Jouko Kumpula

Ecological Applications

Appendix S3. Population-level effect, group -level standard deviation, and group -level correlation parameters, and Smoothing Spline Hyperparameters

Population-level effect parameters (Table S1), Group -level standard deviation parameters (Table S2), Group -level correlation parameters (Table S3), and Smoothing Spline Hyperparameters (Table S4) of Bayesian hierarchical linear regression model used for analyzing the data set on the changes of lichen cover, lichen height, shrub cover and shrub height between 2008-2018 in northernmost Finland.

Table S1. Population-level effect parameters

Response	Parameter	Level	Estimate	Est.Error	l-95% CI	u-95% CI
Lichen Cover Change	Intercept		0.118	0.103	-0.088	0.314
Lichen Cover Change	Seasonal range	Summer	-0.094	0.135	-0.356	0.175
Lichen Cover Change	Seasonal range	Winter	-0.686	0.195	-1.070	-0.306
Lichen Cover Change	Lichen Cover 2008		-0.799	0.012	-0.821	-0.776
Lichen Cover Change	Tree canopy pct 2018		0.001	0.021	-0.041	0.043
Lichen Cover Change	Moss Cover Change		-0.073	0.008	-0.089	-0.057

Lichen Cover Change	Rainy days		0.015	0.025	-0.034	0.063
Lichen Cover Change	GDD5		-0.011	0.020	-0.050	0.028
Lichen Cover Change	Seasonal range: Summer × Rainy days		-0.047	0.031	-0.109	0.014
Lichen Cover Change	Seasonal range: Winter × Rainy days		0.067	0.030	0.008	0.126
Lichen Cover Change	Seasonal range: Summer × GDD5		0.056	0.026	0.003	0.107
Lichen Cover Change	Seasonal range: Winter × GDD5		0.091	0.040	0.012	0.170
Lichen Cover Change	Reindeer density × Seasonal range	All	0.040	0.149	-0.209	0.391
Lichen Cover Change	Reindeer density × Seasonal range	Summer	-0.101	0.184	-0.460	0.305
Lichen Cover Change	Reindeer density × Seasonal range	Winter	-0.155	0.268	-0.807	0.379
**Response**	**Parameter**	**Level**	**Estimate**	**Est.Error**	**l-95% CI**	**u-95% CI**
Lichen Height Change	Intercept		0.329	0.509	-0.664	1.330
Lichen Height Change	Seasonal range	Summer	0.673	0.625	-0.571	1.896
Lichen Height Change	Seasonal range	Winter	0.346	0.775	-1.168	1.869
Lichen Height Change	Lichen height 2008		-0.833	0.015	-0.862	-0.803
Lichen Height Change	Tree canopy pct 2018		0.315	0.131	0.060	0.574
Lichen Height Change	Moss Cover Change		0.084	0.062	-0.037	0.206
Lichen Height Change	Rainy days		0.061	0.140	-0.214	0.332
Lichen Height Change	GDD5		0.091	0.106	-0.120	0.297
Lichen Height Change	Seasonal range: Summer × Rainy days		-0.219	0.172	-0.559	0.115
Lichen Height Change	Seasonal range: Winter × Rainy days		0.204	0.172	-0.126	0.551
Lichen Height Change	Seasonal range: Summer × GDD5		0.009	0.145	-0.276	0.299
Lichen Height Change	Seasonal range: Winter × GDD5		-0.163	0.186	-0.527	0.201

Lichen Height Change	Reindeer density × Seasonal range	All	-0.306	0.490	-1.437	0.624
Lichen Height Change	Reindeer density × Seasonal range	Summer	-0.042	0.607	-1.085	1.337
Lichen Height Change	Reindeer density × Seasonal range	Winter	-0.551	0.635	-1.789	0.961
**Response**	**Parameter**	**Level**	**Estimate**	**Est.Error**	**l-95% CI**	**u-95% CI**
Shrub Cover Change	Intercept		0.003	0.117	-0.227	0.231
Shrub Cover Change	Pasture range	Summer	0.356	0.155	0.050	0.659
Shrub Cover Change	Pasture range	Winter	0.379	0.253	-0.114	0.869
Shrub Cover Change	Shrub Cover 2008		-0.766	0.014	-0.793	-0.739
Shrub Cover Change	Tree canopy pct 2018		-0.081	0.027	-0.134	-0.028
Shrub Cover Change	Moss Cover Change		-0.166	0.014	-0.192	-0.140
Shrub Cover Change	Rainy days		0.036	0.030	-0.021	0.095
Shrub Cover Change	GDD5		0.026	0.022	-0.018	0.069
Shrub Cover Change	Seasonal range: Summer × Rainy days		-0.071	0.038	-0.147	0.002
Shrub Cover Change	Seasonal range: Winter × Rainy days		0.050	0.040	-0.027	0.128
Shrub Cover Change	Seasonal range: Summer × GDD5		-0.008	0.031	-0.069	0.052
Shrub Cover Change	Seasonal range: Winter × GDD5		-0.114	0.052	-0.215	-0.011
Shrub Cover Change	Reindeer density × Seasonal range	All	0.062	0.130	-0.161	0.363
Shrub Cover Change	Reindeer density × Seasonal range	Summer	-0.050	0.190	-0.441	0.323
Shrub Cover Change	Reindeer density × Seasonal range	Winter	0.157	0.836	-1.515	1.781
**Response**	**Parameter**	**Level**	**Estimate**	**Est.Error**	**l-95% CI**	**u-95% CI**
Shrub Height Change	Intercept		-4.419	1.212	-6.793	-2.037
Shrub Height Change	Seasonal range	Summer	-0.076	0.898	-1.862	1.664

Shrub Height Change	Seasonal range	Winter	0.501	0.946	-1.321	2.366
Shrub Height Change	Shrub height 2008		-0.830	0.011	-0.852	-0.808
Shrub Height Change	Tree canopy pct 2018		1.731	0.330	1.076	2.373
Shrub Height Change	Moss Cover Change		-0.038	0.156	-0.343	0.269
Shrub Height Change	Rainy days		0.277	0.314	-0.333	0.895
Shrub Height Change	GDD5		1.549	0.294	0.967	2.123
Shrub Height Change	Seasonal range: Summer × Rainy days		-0.259	0.389	-1.019	0.498
Shrub Height Change	Seasonal range: Winter × Rainy days		-0.081	0.384	-0.837	0.678
Shrub Height Change	Seasonal range: Summer × GDD5		0.306	0.331	-0.337	0.964
Shrub Height Change	Seasonal range: Winter × GDD5		0.037	0.346	-0.634	0.729
Shrub Height Change	Reindeer density × Seasonal range	All	-0.176	0.965	-2.073	1.667
Shrub Height Change	Reindeer density × Seasonal range	Summer	-0.044	0.936	-1.860	1.788
Shrub Height Change	Reindeer density × Seasonal range	Winter	-0.565	0.985	-2.329	1.431

Table S2. Group -level standard deviation parameters (SD)

Response	Parameter	Estimate	Est.Error	l-95% CI	u-95% CI
Lichen Cover Change	Intercept	0.077	0.003	0.071	0.083
Lichen Height Change	Intercept	0.464	0.019	0.428	0.502
Shrub Cover Change	Intercept	0.090	0.004	0.082	0.098
Shrub Height Change	Intercept	1.231	0.051	1.134	1.334

Table S3. Group -level correlation parameters (COR).

Response1	Response2	Estimate	Est.Error	l-95% CI	u-95% CI
Lichen Cover Change_Intercept	Lichen Height Change_Intercept	0.3764	0.04585	0.28498	0.46336
Lichen Cover Change_Intercept	Shrub Cover Change_Intercept	-0.2046	0.05207	-0.3064	-0.1022
Lichen Height Change_Intercept	Shrub Cover Change_Intercept	0.11502	0.05644	0.00362	0.22402
Lichen Cover Change_Intercept	Shrub Height Change_Intercept	-0.2693	0.0473	-0.3614	-0.1747
Lichen Height Change_Intercept	Shrub Height Change_Intercept	0.25658	0.05142	0.15417	0.35573
Shrub Cover Change_Intercept	Shrub Height Change_Intercept	0.70185	0.03742	0.62363	0.77074

Table S4. Smoothing Spline Hyperparameters

Response	Parameter1	Parameter2	Level	Estimate	Est.Error	l-95% CI	u-95% CI
Lichen Cover Change	Reindeer density	Seasonal rotation	All-Year	0.157	0.113	0.014	0.446
Lichen Cover Change	Reindeer density	Seasonal rotation	Summer	0.155	0.114	0.019	0.440
Lichen Cover Change	Reindeer density	Seasonal rotation	Winter	0.109	0.127	0.002	0.446
Lichen Height Change	Reindeer density	Seasonal rotation	All-Year	0.557	0.569	0.012	2.096
Lichen Height Change	Reindeer density	Seasonal rotation	Summer	0.494	0.596	0.013	2.197
Lichen Height Change	Reindeer density	Seasonal rotation	Winter	0.526	0.793	0.008	3.005
Shrub Cover Change	Reindeer density	Seasonal rotation	All-Year	0.110	0.113	0.003	0.405
Shrub Cover Change	Reindeer density	Seasonal rotation	Summer	0.134	0.085	0.038	0.352
Shrub Cover Change	Reindeer density	Seasonal rotation	Winter	0.996	0.432	0.383	2.058
Shrub Height Change	Reindeer density	Seasonal rotation	All-Year	5.391	3.174	0.901	13.046
Shrub Height Change	Reindeer density	Seasonal rotation	Summer	2.547	1.295	0.526	5.643
Shrub Height Change	Reindeer density	Seasonal rotation	Winter	1.055	0.952	0.057	3.570
